# Expression Analysis of the *PITX2* Gene and Associations between Its Polymorphisms and Body Size and Carcass Traits in Chickens

**DOI:** 10.3390/ani9121001

**Published:** 2019-11-20

**Authors:** Haiyue Cao, Xinyang Dong, Haiguang Mao, Ningying Xu, Zhaozheng Yin

**Affiliations:** Animal Science College, Zhejiang University, Zijingang Campus, Hangzhou 310058, China; 11817038@zju.edu.cn (H.C.); sophiedxy@zju.edu.cn (X.D.); maohaiguang@163.com (H.M.); nyxu@zju.edu.cn (N.X.)

**Keywords:** PITX2 gene, single nucleotide polymorphisms, body size and carcass traits, association, chickens

## Abstract

**Simple Summary:**

The Wuliang Mountain Black-bone chicken is a Chinese indigenous breed with good meat quality and strong resistance to disease. Like most of the other Chinese domestic breeds, it has a much slower early growth rate compared with foreign chicken breeds. Therefore, the genetic selection of body size and carcass traits is still the focus of Chinese indigenous chicken breeding. The paired-like homeodomain transcription factor 2 (*PITX2*) gene, an important transcription factor, plays an important role during the development of the eye, heart, skeletal muscle and other tissues in mammals. In chicken, the *PITX2* gene affects the late myogenic differentiation of the limb. The objectives of this study were to detect the expression of the *PITX2* gene and analyze the associations between the polymorphisms in the exons of the *PITX2* gene and body size as well as carcass traits in chickens. The results could contribute to Chinese chicken breeding based on marker assisted-selection.

**Abstract:**

*PITX2* is expressed in and plays an important role in myocytes of mice, and it has effects on late myogenic differentiation in chickens. However, the expression profile and polymorphisms of *PITX2* remain unclear in chickens. Therefore, the aim of the present study was to detect its expression and investigate single nucleotide polymorphisms (SNPs) within its exons and then to evaluate whether these polymorphisms affect body size as well as carcass traits in chickens. The expression analysis showed that the expression level of chicken *PITX2* mRNA in the leg muscle and hypophysis was significantly higher (*p <* 0.01) than those in other tissues. The results of polymorphisms analysis identified two SNPs (i.e., g.9830C > T and g.10073C > T) in exon 1 and 10 SNPs (i.e., g.12713C > T, g.12755C > T, g.12938G > A, g. 3164C > T, g.13019G > A, g.13079G > A, g.13285G > A, g.13335G > A, g.13726A > G and g.13856C > T) in exon 3, including four novel SNPs (i.e., g.9830C > T, g.12713C > T, g.12938G > A and g.13856C > T). In the loci of g.10073C > T and g.12713C > T, chickens with the CT genotype had the highest (*p* < 0.05 or *p* < 0.01) breast depth and breast angle, respectively. For the locus of g.13335G > A, chickens with the GG genotype had the highest (*p* < 0.05 or *p* < 0.01) breast angle and shank circumference. For the locus of g.13726A > G, chickens with the GG genotype had the highest breast width, fossil keel bone length and shank circumference. The locus of g.12713A > G had significant effects on the PITX2 mRNA expression level in leg muscle. The H1H7 diplotype showed the highest shank circumference, and the H2H8 diplotype showed the highest breast muscle rate. The present research suggested that polymorphisms of the exons of the *PITX2* gene were significantly associated with the body size and carcass traits of Wuliang Mountain Black-bone chickens and the *PITX2* gene could be a potential candidate gene for molecular marker-aided selection in Wuliang Mountain Black-bone chickens and other chicken breeds.

## 1. Introduction

Wuliang Mountain Black-bone (WLMB) chicken is a Chinese indigenous breed with good meat quality, strong resistance to disease and medicinal and health-promoting values [1]. Like most of the other Chinese domestic breeds, it has a much slower early growth rate than foreign chicken breeds in which the selection for a fast growth rate has led to major welfare problems, such as musculoskeletal disorders, myopathies and organ failures [2,3,4]. Therefore, the selection of body size and carcass traits is still the focus of Chinese indigenous chicken breeding. Recently, the identification of DNA molecular markers related to quantitative traits based on candidate genes has become an important means of marker-assisted breeding to improve body size and carcass traits [5]. Paired-like homeodomain transcription factor 2 (PITX2) is a member of the bicoid-like homeobox transcription factor family [6], which has a homeobox-2 domain and an OAR domain (15-aa) [7]. The homeobox-2 domain can combine specifically with DNA through its helix-turn-helix (HTH) structure, and the OAR domain is where PITX2 interacts with pituitary homeobox 1.

In the past two decades, many researchers have demonstrated that the *PITX2* gene is expressed in, and plays an important role during, the development of various tissues in mice, such as the heart, lung and dental germ [8,9,10,11]. In the myocytes of mice, the expression of myogenic regulatory factors (MRFs) might be regulated by the *PITX2* gene [12]. PITX2-overexpression causes significant down-regulation of MyoD (an MRF) and an up-regulation of paired box factor 3 gene [13]. A subset of microRNA regulated by *PITX2* also has profound effects on myoblast proliferation [14]. Moreover, it has been reported that the PITX2 protein can activate the Wnt signaling pathway and induce cell proliferation [15,16,17].

In chicken, the expression pattern of *PITX2* is highly similar to that of mouse during pituitary development [18,19,20], and the *PITX2* gene affects the late myogenic differentiation of the limb in the chick embryo [21]. These results suggested that the *PITX2* gene is associated with the development of myocytes. 

Recently, polymorphism studies of the *PITX2* gene were carried out on humans and mainly focused on its associations with diseases [22,23,24]. In livestock, polymorphisms of the *PITX2* gene have been reported to be related to the milk traits of dairy goats and the meat quality and growth of pigs [25,26,27]. However, the expression profile and polymorphisms of *PITX2* remain unclear in poultry. Therefore, the objectives of this study were to detect the expression of the *PITX2* gene and analyze the associations between the polymorphisms in the exons of the *PITX2* gene and body size as well as carcass traits in chickens. The *PITX2* gene of chickens is 4112bp long with three exons and is located on chromosome 4. The results of this study could contribute to functional research on the *PITX2* gene and chicken breeding based on marker-assisted selection.

## 2. Materials and Methods 

The present experiment was conducted following Chinese guidelines for animal welfare and was approved by the animal welfare committee of Zhejiang University (Approval Number: 12969).

### 2.1. Experimental Animals and Design

In the present study, Wuliang Mountain Black-bone (WLMB) chickens were used. These chickens have feathered feet, green ear lobes and black bones, along with the presence of massive dermal and visceral pigmentation. According to the Standards of Agricultural Industry of the People’s Republic of China (NY/T 828-2004), the age of 300 days is a typical period to reflect the breed characteristics of poultry. Four hundred (300-day-old) female WLMB chickens, randomly selected from Wuliang Mountain Black-bone Chicken Professional Cooperatives of Long Street (Jingdon, China), were measured for body size and carcass traits for the association analysis with polymorphisms of the *PITX2* gene. A total of 11 different tissues (heart, liver, spleen, lung, kidney, breast muscle, leg muscle, muscular stomach, glandular stomach, hypothalamus and hypophysis) were separately isolated from 10 chickens (randomly selected from 400 WLMB chickens) which were used for analyzing *PITX2* mRNA expression in different tissues. After single nucleotide polymorphisms (SNPs)-traits association and tissues relative expression analysis, 10 individuals for each genotype of each SNP (a total of 120 WLMB chickens were randomly selected from 400 WLMB chickens) associated with body size or carcass traits were selected, analyzing the *PITX2* mRNA relative expression in their leg muscles.

The body size traits, including body slope length (BSL), breast width (BW), breast depth (BD), breast angle (BA), fossil bone length (FBL), pelvis width (PW), shank length (SL) and shank circumference (SC) were measured before slaughter. Live weight (LW) was also measured before slaughter, and then carcass weight (CW), semi-eviscerated weight (SEW), eviscerated weight (EW), breast muscle weight (BMW) and abdominal fat weight (AFW) were measured on the carcass. The ratio of CW, SEW and EW to LW was calculated as slaughter rate (SR), semi-eviscerated rate (SER), eviscerated rate (ER), respectively. The ratios of BMW to EW were calculated as breast muscle rate (BMR). The ratios of AFW to the sum of EW and AFW were calculated as abdominal fat rate (AFR). All of the 8 body size traits and 11 carcass traits were evaluated in accordance with the Standards of the agricultural industry of the People’s Republic of China (NY/T 823-2004).

### 2.2. DNA and RNA Extraction, cDNA Synthesis

Blood samples were collected from the jugular vein and stored at −20 °C (with EDTA) until genomic DNA extraction was performed. Genomic DNA was extracted using a Genome DNA Extraction Kit (TIANGEN, Beijing, China) under the guidance of the instruction manual. 

Tissues were frozen immediately in liquid nitrogen after being separated and stored at −80 °C. Total RNA was extracted using TRIzol A^+^ Total RNA reagent (TIANGEN, Beijing, China). The amount and purity (OD260/OD280 ratio > 1.9) of the extracted RNA were measured by the NanoDropND2000 spectrophotometer (Thermo Fisher Scientific, Waltham, MA, USA), and the quality of the obtained RNA was checked by electrophoresis. The PrimeScript RT Reagent Kit with gDNA Eraser (Takara, Japan) was used to convert RNA to cDNA following the manufacturer’s protocol.

### 2.3. PCR Amplification and Sequencing

Primer pairs (Table 1) of the 3 exons of the *PITX2* gene were designed using Primer Premier 5.0 software (Premier Biosoft International, Palo Alto, CA, USA) based on the chicken *PITX2* gene sequences (Gene ID: 395862). Primers were synthesized by Tsingke Biotech Co., Ltd. (Hangzhou, China). PCR amplification was performed using a PCR instrument (Eppendorf, Germany) with a 50 μL PCR mixture including 25 μL of 2× Taq PCR Master Mix (including Mg2+, dNTP and Taq DNA Polymerase) from Tsingke Biotech Co., Ltd. (Hangzhou, China), 2 μL of each primer (10 μmol/L), 2 μL DNA template and 21 μL double-distilled water (Sangon Biotech, Shanghai, China).

The PCR amplifications were performed with an initial denaturation cycle at 94 °C for 5 min followed by 35 cycles of 94 °C for 30 s, 45 s at the annealing temperature and extension at 72 °C for 90 s, and ending with an extension cycle at 72 °C for 10 min. The PCR products were checked by 0.8% agarose gel electrophoresis. Next, the PCR samples obtained were sequenced using Sanger sequencing by Tsingke Biotech Co., Ltd. (Hangzhou, China).

### 2.4. Relative Expression of PITX2 mRNA

The primer pairs (Table 1) of the *PITX2* mRNA (Transcript: PITX2-202 ENSGALT00000075211.2) for real-time fluorescent quantitative PCR were designed using Primer-BLAST (https://www.ncbi.nlm.nih.gov/tools/primer-blast/) based on the chicken *PITX2* mRNA sequences (NM_205010.1). Primers were synthesized by Tsingke Biotech Co., Ltd. (Hangzhou, China).

Real-time PCR was performed using a StepOnePlus Real-Time PCR System (Applied Biosystems, Foster City, CA, USA) with a 20 μL PCR mixture including 2 μL of cDNA, 10 μL of 2 × SYBR Premix Ex TaqII, 0.4 μL ROX Reference Dye (50×) 0.8 μL of forward primer, 0.8 μL of reverse primer and 6 μL of nuclease-free water. The 2× SYBR Premix Ex TaqII, ROX Reference Dye (50×) and nuclease-free water were contained in the TB Green Premix Ex Taq TM II (Takara, Dalian, China). Reactions were incubated in a 96-well optical plate (Applied Biosystems, American) at 95 °C for 30 s, followed by 40 cycles of 95 °C for 5 s, and 60 °C for 30 s. Each sample was analyzed in triplicate. The expression levels of the mRNA were normalized to the mRNA expression of chicken *β-actin* and were calculated using the 2^−ΔΔ*C*t^ method [28].

### 2.5. Statistical Analysis

MEGA 6.0 was used to align the PITX2 amino acid sequences of 12 species (*Gallus gallus*: NP_990341.1, *Homo sapiens*: NP_000316.2, *Bos Taurus*: NP_001091460.1, *Oryctolagus cuniculus*: XP_017202834.1, *Pan troglodytes*: XP_001141234.1, *Sus scrofa*: NP_001193364.1, *Mus musculus*: NP_001035967.1, *Capra hircus*: NP_001301188.1, *Nothoprocta perdicaria*: XP_025906661.1, *Parus major*: XP_015479219.1, *Rattus norvegicus*: NP_001035970.1 and *Anas platyrhynchos*: XP_027312253.1), and then used to construct a neighbor-joining phylogenetic tree. Confidence values for the nodes were determined using the bootstrap test by 1000 replicates [29].

Sequences were analyzed with SeqMan II version 5.01 (DNAStar Inc., Madison, WI, USA). Genotype frequency, allele frequency and diversity parameters were calculated and the deviations from Hardy–Weinberg equilibrium (*HWE*) were checked. HaploView was used to analyze linkage disequilibrium (*LD*). Haplotypes and diplotypes were analyzed using PHASE 2.1, which implements a Bayesian statistical method for reconstructing haplotypes from population genotype data.

The general linear model (GLM) was separately implemented on each SNP and diplotype using statistical software SPSS 20.0 (SPSS, Chicago, IL, USA). The linear model used was as follows:

$$Y_{ijk}=\mu +G_{i}+B_{j}+e_{ijk}$$

where, $Y_{ijk\;}$= the phenotypic value of traits, μ = the least squares mean of the population, $G_{i}$ = effect of genotypes (*i* = 1, 2 or 3) or diplotypes (*i* = 1, 2, 3, 4, 5, 6, 7 or 8), $B_{j}$ = effect of birthdate (*j* = 1, 2, or 3) and $e_{ijk}$ = random residual error. Multiple comparison results of the genotypes and diplotypes were corrected by Bonferroni correction.

The additive and dominance effects of the *PITX2* gene were estimated as follows [30]:

Additive effect = (AA − BB)/2(1)

Dominance effect = AB − (AA + BB)/2(2)

where AA, AB and BB were the least-squares means of the genotype AA, AB and BB groups, respectively. The AA genotype was a homozygous genotype for the two bases located before the ‘>’ of the SNPs, while BB was a homozygous genotype for the two bases situated after the ‘>’ of the SNPs. For example, the AA, AB and BB genotypes of the g.9830C > T locus in the *PITX2* gene means the genotypes of CC, CT and TT.

The relative expression data were checked for normality by the Kolmogorov–Smirnov test in statistical software SPSS v20.0 (IBM, Chicago, IL, USA), and logarithmic transformation (log2) was used to correct the non-normal distribution. Then, one-way ANOVA was used to analyze the relative expression of the *PITX2* gene among groups. Multiple comparison results of the groups were corrected by Bonferroni correction.

## 3. Results

### 3.1. Phylogenetic Relationships of PITX2 Proteins among 12 Species

**Figure 1:**
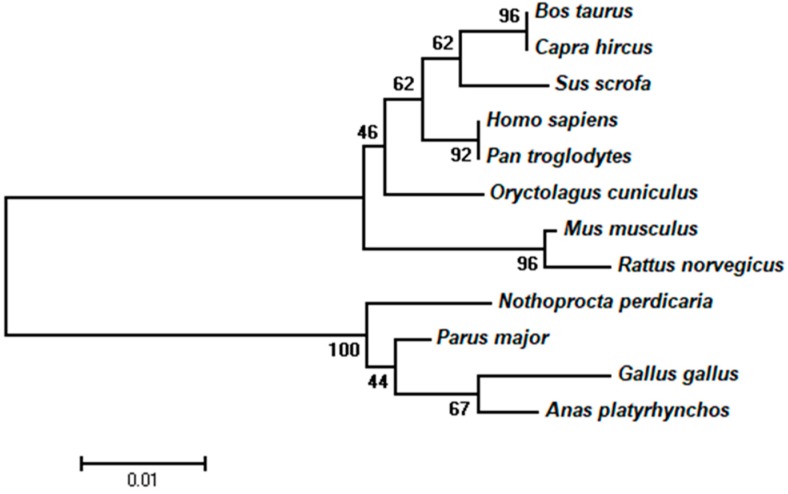
Phylogenetic tree constructed based on the PITX2 amino acid sequence of 12 species. Branches were labeled with species’ Latin names. The number above each branch represented bootstrap values.

As shown in Figure 1, the PITX2 proteins of all 12 species fell into two subgroups. All of the mammal sequences clustered into one subgroup and the sequences from the four avian species (*Gallus gallus*, *Nothoproctaperdicaria*, *Parus major* and *Anas platyrhynchos*) clustered into another subgroup.

### 3.2. Relative Expression Levels of Chicken PITX2 mRNA in 11 Tissues

**Figure 2:**
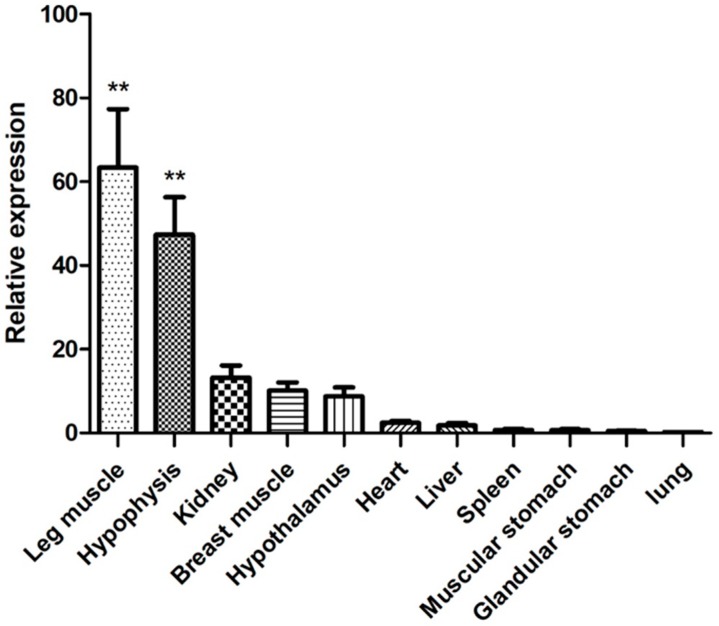
Relative expression levels of chicken *PITX2* mRNA in 11 tissues with the average ΔCt value of heart as the calibrator. Data were shown as the mean ± SEM for 10 chickens. Columns “**” showed highly significant difference (*p* < 0.01).

The relative expression results showed that chicken *PITX2* mRNA was widely expressed in the heart, liver, spleen, lung, kidney, breast muscle, leg muscle, muscular stomach, glandular stomach, hypothalamus and hypophysis, but the level of expression was regulated in a tissue-specific fashion. The expression level of chicken *PITX2* mRNA was significantly higher (*p <* 0.01) in the leg muscle and hypophysis than in other tissues (Figure 2).

### 3.3. Genetic Polymorphisms

The results of sequence analysis showed that g.9830C > T and g.10073C > T in exon 1 and g.12713C > T, g.12755C > T, g.12938G > A, g.12961C > T, g.13019G > A, g.13079G > A, g.13285G > A, g.13335G > A, g.13726A > G and g.13856C > T in exon 3 were found in the chicken *PITX2* gene, including four novel SNPs (i.e., g.9830C > T in exon 1, and g.12713C > T, g.12938G > A and g.13856C > T in exon 3). There was no variation found in exon 2. As shown in Table 2, no mutations led to amino acid changes.

### 3.4. Genotypic Frequencies, Allelic Frequencies and Diversity Parameter

Genotypic frequency, allele frequency and diversity parameters were displayed in Table 3. All SNPs exhibited three genotypes, except g.13856C > T. The dominant alleles of all SNPs were non-mutation alleles. The *χ*2-test showed that the genotypic distributions of different variants were consistent with HWE (*p* > 0.05), except g.9830C > T (*p* < 0.05). Moreover, the data of polymorphism information content (PIC) indicated that g.13335G > A and g.13726A > G were moderately polymorphic while the rest were lowly polymorphic. The locus of g.9830C > T was not used in further analysis since it deviated from the *HWE*. After excluding the genotypes with less than 10 individuals, the SNPs with three genotypes (g.10073C > T, g.12713C > T, g.13335G > A and g.13726A > G) were used for further analysis of the association between the SNPs and body size as well as carcass traits.

### 3.5. Analysis of LD Coefficient, Haplotype and Diplotype

**Figure 3:**
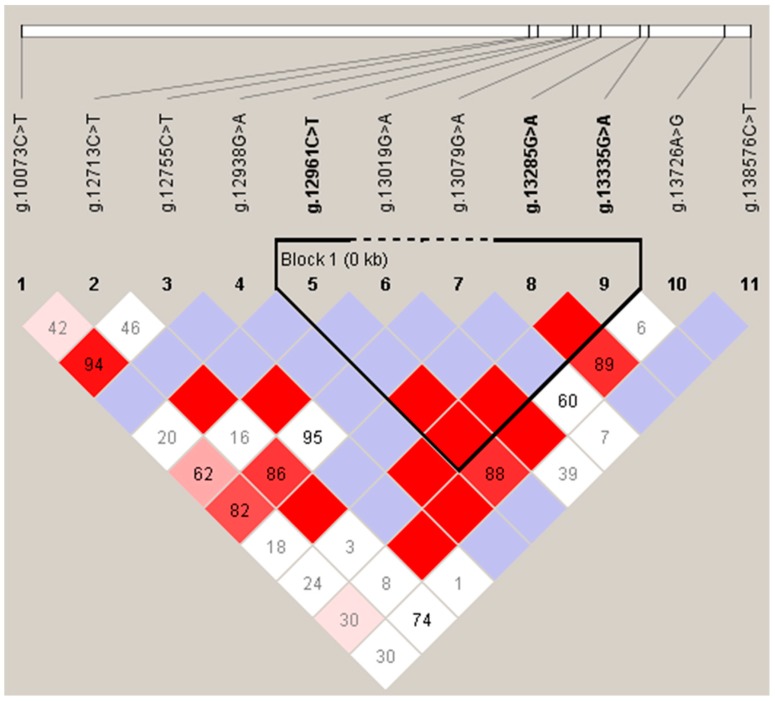
Analysis of blocks with 11 SNPs in the *PITX2* gene in Wuliang Mountain Black-bone chickens. The values in the squares indicate the paired linkage disequilibrium (*LD*) values (*D’*) of the SNPs. When *D’* = 1, the values will not be displayed. The redder the square, the stronger the *LD*. The haplotype block was defined using the default setting of the Haploview software.

The results of *LD* analysis indicated that g.12961C > T, g.13019G > A, g.13079G > A, g.13285G > A, and g.13335G > A showed strong *LD*, constructing a 374 bp block 1 (Figure 3). As shown in Table 4, nine haplotypes (H1-H9) were formed based on block 1, where the H1 haplotype showed the highest frequency, followed by H2 and H7. In the diplotype study, eight diplotypes were formed based on the nine haplotypes, where the frequency of H1H2 was the highest followed by H1H1. The diplotypes with fewer than 10 individuals were excluded.

### 3.6. Association of Gene Polymorphisms with Body Size and Carcass Traits

The association of *PITX2* gene SNPs with body size and carcass traits of WLMB chickens are shown in Table 5 and Table 6. For the locus of g.10073C > T, BD in chickens with the CT genotype was significantly higher than those with the CC (*p* < 0.05) and TT genotypes (*p* < 0.01). For the locus of g.12713C > T, chickens with the CT genotype had a higher BA than those with the CC genotype (*p* < 0.01). For the locus of g.13335G > A, chickens with the GG genotype had a higher BA than those with the GA genotype (*p* < 0.01), and a higher SC than those with GA (*p* < 0.05) and AA genotypes (*p* < 0.01). For the locus of g.13726A > G, chickens with the GG genotype had higher FBL and SC than those with the AA genotype (*p* < 0.05), and a higher BW than those with AA (*p* < 0.01) and AG genotypes (*p* < 0.05). Moreover, g.10073C > T had an over-dominant effect on BD, and g.12713C > T and g.13335G > A had an over-dominant effect on BA. In contrast, for g.13335G > A on SC, and g.13726A > G on BW, FBL and SC, the additive effect was bigger than the dominance effect, whereas they had no association with carcass traits (Table 6). Table 7 and Table 8 show the association of diplotypes with body size and carcass traits. SC in chickens with the H1H7 diplotype was significantly higher than those with the H1H4 diplotype (*p* < 0.01) and chickens with the H2H8 diplotype had a higher BMR than those with the H1H2 and H1H8 diplotypes.

### 3.7. PITX2 mRNA Relative Expression with Different Genotypes in Leg Muscle

**Figure 4:**
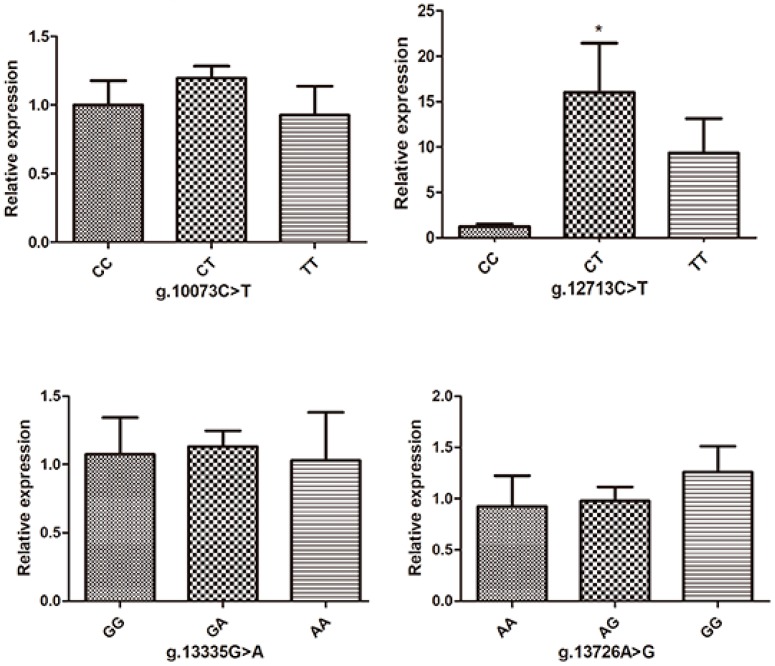
Relative expression levels of the chicken *PITX2* mRNA in leg muscle with different genotypes in the loci of g.10073C > T, g.12713C > T, g.13335G > A and g.13726A > G with the average ΔCt value of CC, CC, GG and AA genotype as the calibrator, respectively. Data were shown as the mean ± SEM for 10 chickens of each genotype. The column with “*” showed a significant difference (*p* < 0.05).

Since the highest *PITX2* mRNA relative expression was in the leg muscle, we selected it to detect expression levels for different genotypes of the loci (g.10073C > T, g.12713C > T, g.13335G > A and g.13726A > G) that were significantly associated with body size traits. The results showed that only the genotype of locus g.12713C > T was significantly associated with the expression level of the *PITX2* gene. As shown in Figure 4, for the locus of g.12713C > T, leg muscles with the CT genotype had higher (*p* < 0.05) *PITX2* mRNA expression level than those with CC and TT genotypes.

## 4. Discussion

Based on the construction of a neighbor-joining phylogenetic tree in which the PITX2 amino acid sequences of 12 species were aligned, the result showed that the PITX2 amino acid sequences were relatively conserved and the SNPs detected in the present study occurred at conserved regions, which indicated the function of the *PITX2* gene might be similar across the 12 species. As shown in the phylogenetic tree, chicken (*Gallus gallus*) and mallard (*Anas platyrhynchos*) were grouped, indicating that the function of the *PITX2* gene in chicken was most similar to that in mallard.

In our study, *PITX2* mRNA was expressed widely in 11 tissues of WLMB chickens which was the same as in other animals [32]. Although the study of Abu-Elmagd et al. did not detect an effect of *PITX2* on early limb myogenesis in the chick embryo, they found the late myogenic differentiation in the limb was affected by *PITX2* [21]. Moreover, several studies have confirmed that the expression of the *PITX2* gene can activate the LIM, POU and SIX families to regulate the development and function of the hypophysis [33]. *PITX2* mRNA was also detected during the early development of the hypophysis in the chick [34]. These studies showed that the *PITX2* gene might play an important role in the development of chicken limb muscles and the hypophysis. In the present study, the expression level of the *PITX2* gene in the leg muscle and hypophysis was the highest, which was consistent with these prior reports.

In the current research, 12 SNPs were found in the exons of the chicken *PITX2* gene, including four novel SNPs that have not been previously recorded by NCBI. According to the results of the *χ*2 test, g.9830C > T was not in agreement with *HWE*, which might be explained by the artificial selection in the breed [27]. All mutations were synonymous, demonstrating that the sequence of the PITX2 amino acids seemed to be conserved [35]. It is worth noting that silent mutations can also affect gene function and phenotype by regulating the stability of the mRNA or inducing alternative splicing events [36,37,38]. Synonymous mutations change the tRNA that transports the same amino acid, and abundances of different tRNAs are different, which will influence the transcription efficiency [39]. Moreover, the function of microRNA is to regulate the mRNA level and protein expression by binding to the 3′UTR of mRNA [40]. Thus, silent mutations could not be ignored.

Polymorphisms of the *PITX2* gene are significantly related to the milk traits and meat quality of dairy goats and pigs, respectively, and might contribute to the improvement of these traits [26,27]. Significant effects of the polymorphisms of the *PITX2* gene on chicken body size and carcass traits were detected in the current study. The results showed that four SNPs (g.10073C > T, g.12713A > G, g.13335G > A and g.13726A > G) were associated with the body size traits of WLMB chickens. The locus of g.12713A > G had significant effects on the *PITX2* mRNA expression level in leg muscle. Three SNPs of the *PITX2* gene also had significant effects on goat growth traits and mRNA expression levels [41]. The four mutations associated with body size traits in this study were silent. The molecular mechanism of how the four silent mutations affect the phenotype still needs further research. Although there were no SNPs associated with the carcass traits, the diplotypes had a significant effect on BMR. All of the above demonstrate that polymorphisms of the chicken *PITX2* gene are associated with the body size and carcass traits of WLMB chickens.

## 5. Conclusions

In summary, the present study showed that the expression level of chicken *PITX2* mRNA in the leg muscle and hypophysis was the highest, and polymorphisms of the *PITX2* gene were significantly associated with the body size and carcass traits of Wuliang Mountain Black- bone chickens, which indicated that the *PITX2* gene could be a potential candidate gene for molecular marker-aided selection in chicken breeding.

## Figures and Tables

**Table 1:** Primers sequences for amplifying the exons and detecting the expression levels of paired-like homeodomain transcription factor 2 (PITX2) in chicken.

Primer	Sequence (5′~3′)	Length (bp)	Annealing Temperature (°C)
*PITX2-1*	F: GGGCACACGCGCTCCTT	430	60
	R: CTCGCCCTCTACAACCGAT		
*PITX2-2*	F: AGCGGTAACGGACAGCAAC	717	60
	R: GCCAATGGTTTCCGTAGC		
*PITX2-3*	F: CAGCGTTCTTCCCTGTGGT	1538	60
	R: CCGAAAAAGTGCGGCGTT		
*PITX2-m*	F: GTCCTCTCGCCGATGAGTTG	212	60
	R: GCTTATTATTCCCGGCTCCCA		
*β-actin*	F: ACGTCGCACTGGATTTCGAG	282	60
	R: TGTCAGCAATGCCAGGGTAC		

*PITX2*-1, *PITX2*-2 and *PITX2*-3: the primer pairs of the exon 1, exon 2 and exon 3 of chicken *PITX2* gene in PCR amplification, respectively. *PITX2-m* and *β-actin* [28]: the primer pairs of chicken *PITX2* mRNA and chicken *β-actin* mRNA in real-time fluorescent quantitative PCR, respectively.

**Table 2:** Alleles and genotypes of single nucleotide polymorphisms (SNPs) in the exons of the *PITX2* gene and the amino acid mutations.

SNPs	Genotypes	Mutation Sites			Amino Acid Mutations
		Exon of *PITX2*	*PITX2*	Chromosome	
g.9830C > T	CC, CT, TT	1	9830	57794861	---
g.10073C > T	CC, CT, TT	1	10073	57795104	Serine
g.12713C > T	CC, CT, TT	3	12713	57797744	Asparagine
g.12755C > T	CC, CT, TT	3	12755	57797786	Aspartic acid
g.12938G > A	GG, GA, AA	3	12938	57797969	Serine
g.12961C > T	CC, CT, TT	3	12961	57797992	Serine
g.13019G > A	GG, GA, AA	3	13019	57798050	Alanine
g.13079G > A	GG, GA, AA	3	13079	57798110	Threonine
g.13285G > A	GG, GA, AA	3	13285	57798316	---
g.13335G > A	GG, GA, AA	3	13335	57798366	---
g.13726A > G	AA, AG, GG	3	13726	57798757	---
g.13856C > T	CC, CT	3	13856	57798887	---

SNPs: single nucleotide polymorphism sites. ‘---’: the mutation site located in the non-coding area of mRNA transcribed by the *PITX2* gene.

**Table 3:** Genotypic frequency, allelic frequency and diversity parameter in the exons of the *PITX2* gene in Wuliang Mountain Black-bone chickens.

SNPs	Genotypic Frequency			Allelic Frequency		*p-Value*	*PIC*	*He*	*Ne*
	CC	CT	TT	C	T				
g.9830C > T	0.78 (312)	0.18 (71)	0.04 (17)	0.87 (695)	0.13 (105)	0.0000	0.2020	0.2280	1.2954
g.10073C > T	0.71 (285)	0.25 (100)	0.04 (15)	0.84 (670)	0.16 (130)	0.1030	0.2351	0.2722	1.3740
g.12713C > T	0.73 (292)	0.24 (94)	0.04 (14)	0.85 (678)	0.15 (122)	0.0692	0.2251	0.2585	1.3486
g.12755C > T	0.94 (377)	0.06 (22)	0.00 (1)	0.97 (776)	0.03 (24)	0.2715	0.0565	0.0582	1.0618
	GG	GA	AA	G	A				
g.12938G > A	0.95 (380)	0.05 (19)	0.00 (1)	0.97 (779)	0.03 (21)	0.1565	0.0498	0.0511	1.0539
	CC	CT	TT	C	T				
g.12961C > T	0.74 (295)	0.25 (99)	0.02 (6)	0.86 (689)	0.14 (111)	0.4767	0.2104	0.2390	1.3141
	GG	GA	AA	G	A				
g.13019G > A	0.92 (366)	0.08 (33)	0.00 (1)	0.96 (765)	0.04 (35)	0.7794	0.0802	0.0837	1.0913
g.13079G > A	0.93 (371)	0.07 (28)	0.00 (1)	0.96 (770)	0.04 (30)	0.5445	0.0696	0.0722	1.0778
g.13285G > A	0.73 (293)	0.25 (101)	0.02 (6)	0.86 (687)	0.14 (113)	0.4143	0.2132	0.2426	1.3203
g.13335G > A	0.51 (202)	0.39 (155)	0.11 (43)	0.70 (559)	0.30 (241)	0.1115	0.3324	0.4210	1.7271
	AA	AG	GG	A	G				
g.13726A > G	0.33 (133)	0.48 (190)	0.19 (77)	0.57 (456)	0.43 (344)	0.5352	0.3701	0.4902	1.9616
	CC	CT	TT	C	T				
g.13856C > T	0.97 (386)	0.04 (14)	0.00 (0)	0.98 (786)	0.02 (14)	0.7217	0.0338	0.0344	1.0356

SNPs: single nucleotide polymorphism sites. *p-value*: the *χ*^2^ test of Hardy–Weinberg equilibrium: *p* > 0.05 suggested the population conformed to Hardy–Weinberg equilibrium. *PIC*: polymorphism information content. *PIC* > 0.5 meant highly polymorphic, 0.25 < *PIC* < 0.5 signified moderate polymorphism, *PIC* < 0.25 showed low polymorphism [31]. *He*: heterozygosity. *Ne*: effective number of alleles.

**Table 4:** Haplotypes and diplotypes based on the block 1 and frequencies in Wuliang Mountain Black-bone chickens.

Haplotypes	1	2	3	4	5	Frequency	Diplotypes	Frequency
H1(437)	C	G	G	G	G	0.5463	H1H1(93)	0.2325
H2(143)	C	G	G	G	A	0.1788	H1H2(102)	0.2550
H3(23)	C	G	G	A	G	0.0288	H1H3(19)	0.0475
H4(54)	C	G	G	A	A	0.0675	H1H4(52)	0.1300
H5(1)	C	G	A	G	G	0.0013	H1H7(62)	0.1550
H6(2)	C	G	A	G	A	0.0025	H1H8(15)	0.0375
H7(105)	C	A	A	G	G	0.1313	H2H7(31)	0.0775
H8(29)	T	G	G	G	G	0.0363	H2H8(10)	0.0250
H9(6)	T	A	G	G	G	0.0075		

Diplotypes with fewer than 10 individuals were excluded. 1: g.12961C > T, 2: g.13019G > A, 3: g.13079G > A, 4: g.13285G > A, 5: g.13335G > A.

**Table 5:** Association of four SNPs of the *PITX2* gene with body size traits in Wuliang Mountain Black-bone chickens (*MEAN ± SE*).

SNPs	Genotypes	Body Size Traits							
		BSL (cm)	BW (cm)	BD (cm)	BA (cm)	FBL (cm)	PW (cm)	SL (cm)	SC (cm)
g.10073C > T	CC	21.52 ± 0.09	7.69 ± 0.04	11.10 ± 0.08^b^	55.58 ± 0.63	11.40 ± 0.07	8.46 ± 0.04	10.68 ± 0.06	4.56 ± 0.04
	CT	22.30 ± 0.61	7.65 ± 0.08	11.40 ± 0.11^Aa^	55.62 ± 1.19	11.43 ± 0.10	8.57 ± 0.07	10.81 ± 0.09	4.56 ± 0.06
	TT	21.31 ± 0.39	7.71 ± 0.17	10.48 ± 0.42^Bb^	55.00 ± 2.32	11.07 ± 0.26	8.59 ± 0.12	10.52 ± 0.40	4.60 ± 0.15
	Additive	0.105	−0.01	0.31	0.29	0.165	−0.065	0.08	−0.02
	Dominance	0.885	−0.05	0.61	0.33	0.195	0.045	0.21	−0.02
g.12713C > T	CC	21.67 ± 0.08	7.70 ± 0.04	11.17 ± 0.08	55.09 ± 0.64^B^	11.41 ± 0.06	8.49 ± 0.04	10.77 ± 0.06	4.56 ± 0.04
	CT	21.98 ± 0.67	7.64 ± 0.09	11.11 ± 0.13	57.38 ± 1.05^A^	11.37 ± 0.12	8.50 ± 0.07	10.60 ± 0.11	4.58 ± 0.06
	TT	20.73 ± 0.45	7.52 ± 0.17	10.94 ± 0.39	53.43 ± 3.47	11.10 ± 0.34	8.53 ± 0.14	10.16 ± 0.41	4.57 ± 0.15
	Additive	0.47	0.09	0.115	0.83	0.155	−0.02	0.305	−0.005
	Dominance	0.78	0.03	0.055	3.12	0.115	−0.01	0.135	0.015
g.13335G > A	GG	21.89 ± 0.32	7.68 ± 0.05	11.12 ± 0.09	56.51 ± 0.79^A^	11.37 ± 0.08	8.53 ± 0.06	10.73 ± 0.07	4.62 ± 0.04^Aa^
	GA	21.53 ± 0.12	7.71 ± 0.06	11.19 ± 0.10	54.12 ± 0.85^B^	11.46 ± 0.10	8.44 ± 0.05	10.71 ± 0.08	4.56 ± 0.05^a^
	AA	21.45 ± 0.17	7.58 ± 0.09	11.16 ± 0.16	56.41 ± 1.53	11.26 ± 0.14	8.51 ± 0.09	10.62 ± 0.13	4.30 ± 0.07^Bb^
	Additive	0.22	0.05	−0.02	0.05	0.055	0.01	0.055	0.16
	Dominance	−0.14	0.08	0.05	−2.34	0.145	−0.08	0.035	0.1
g.13726A > G	AA	21.37 ± 0.12	7.61 ± 0.06^Bb^	11.08 ± 0.11	55.34 ± 0.93	11.19 ± 0.09^b^	8.43 ± 0.06	10.55 ± 0.09	4.46 ± 0.05^b^
	AG	21.93 ± 0.33	7.64 ± 0.05^b^	11.26 ± 0.09	55.69 ± 0.77	11.46 ± 0.08	8.50 ± 0.05	10.77 ± 0.07	4.59 ± 0.04
	GG	21.74 ± 0.18	7.89 ± 0.09^Aa^	11.01 ± 0.16	55.66 ± 1.35	11.58 ± 0.14^a^	8.57 ± 0.10	10.84 ± 0.12	4.68 ± 0.07^a^
	Additive	−0.185	−0.14	0.035	−0.16	−0.195	−0.07	−0.145	−0.11
	Dominance	0.375	−0.11	0.215	0.19	0.075	0	0.075	0.02

SNPs: single nucleotide polymorphism sites. BSL, body slope length; BW, breast width; BD, breast depth; BA, breast angle; FBL, fossil bone length; PW, pelvis width; SL, shank length; SC, shank circumference. Means with different lowercase superscripts differed significantly (*p* < 0.05); means with different capital superscripts differed highly significantly (*p* < 0.01); means with the same letter did not differ significantly (*p* > 0.05).

**Table 6:** Association of four SNPs of the *PITX2* gene with carcass traits in Wuliang Mountain Black-bone chickens (*MEAN ± SE*).

SNPs	Genotypes	Carcass traits										
		LW (g)	CW (g)	SR (%)	SEW (g)	SER (%)	EW (g)	ER (%)	BMW (g)	BMR (%)	AFW (g)	AFR (%)
g.10073C > T	CC	1684.64 ± 16.58	1546.01 ± 15.87	91.71 ± 0.18	1339.54 ± 13.64	79.51 ± 0.22	1100.49 ± 10.92	65.40 ± 0.23	167.67 ± 2.27	15.31 ± 0.17	40.22 ± 2.10	3.26 ± 0.15
	CT	1710.27 ± 27.56	1573.41 ± 25.43	92.00 ± 0.24	1367.62 ± 22.71	80.17 ± 0.34	1124.02 ± 18.01	66.00 ± 0.41	172.00 ± 3.87	15.38 ± 0.29	45.88 ± 3.73	3.57 ± 0.25
	TT	1663.27 ± 79.03	1538.80 ± 76.13	92.44 ± 0.45	1336.33 ± 62.09	80.54 ± 0.99	1081.80 ± 44.80	65.44 ± 1.14	164.62 ± 10.04	15.40 ± 0.14	57.77 ± 13.39	4.62 ± 0.87
g.12713C > T	CC	1690.63 ± 16.72	1551.33 ± 15.77	91.73 ± 0.18	1347.98 ± 13.53	79.77 ± 0.21	1107.73 ± 10.75	65.65 ± 0.23	168.47 ± 2.20	15.29 ± 0.16	42.19 ± 2.15	3.39 ± 0.15
	CT	1701.24 ± 27.02	1566.47 ± 25.98	92.01 ± 0.22	1351.97 ± 23.32	79.60 ± 0.38	1108.19 ± 18.64	65.33 ± 0.39	169.91 ± 4.30	15.43 ± 0.33	43.59 ± 3.91	3.43 ± 0.27
	TT	1608.36 ± 71.04	1485.57 ± 72.96	92.15 ± 0.53	1276.07 ± 59.80	79.35 ± 1.07	1044.71 ± 48.00	65.02 ± 1.03	163.21 ± 9.83	15.58 ± 0.88	35.68 ± 8.04	3.02 ± 0.56
g.13335G > A	GG	1700.21 ± 21.18	1561.68 ± 19.91	91.81 ± 0.18	1352.23 ± 17.20	79.56 ± 0.26	1106.17 ± 13.48	65.19 ± 0.27	167.50 ± 2.47	15.30 ± 0.20	44.34 ± 2.75	3.53 ± 0.18
	GA	1678.69 ± 20.96	1540.51 ± 19.92	91.75 ± 0.26	1339.30 ± 17.49	79.91 ± 0.31	1103.82 ± 14.28	65.93 ± 0.34	168.69 ± 3.42	15.29 ± 0.24	40.74 ± 2.81	3.28 ± 0.20
	AA	1685.09 ± 36.49	1553.40 ± 37.01	92.03 ± 0.38	1344.33 ± 30.73	79.74 ± 0.42	1109.53 ± 24.48	65.88 ± 0.42	173.71 ± 5.85	15.63 ± 0.38	38.27 ± 4.78	3.12 ± 0.36
g.13726A > G	AA	1658.77 ± 23.26	1526.53 ± 22.69	91.93 ± 0.26	1321.95 ± 19.69	79.63 ± 0.31	1088.57 ± 15.84	65.66 ± 0.36	166.26 ± 3.33	15.31 ± 0.23	41.15 ± 3.02	3.37 ± 0.21
	AG	1698.42 ± 20.74	1557.01 ± 19.23	91.68 ± 0.21	1351.22 ± 16.91	79.60 ± 0.27	1107.14 ± 13.43	65.31 ± 0.28	169.91 ± 2.83	15.44 ± 0.22	41.85 ± 2.68	3.36 ± 0.19
	GG	1724.47 ± 31.95	1586.68 ± 30.85	91.91 ± 0.27	1377.09 ± 25.52	80.14 ± 0.40	1131.70 ± 20.06	65.96 ± 0.42	169.54 ± 4.25	15.09 ± 0.33	45.34 ± 4.53	3.50 ± 0.29

SNPs: single nucleotide polymorphism sites. LW, live weight; CW, carcass weight; SR, slaughter rate; SEW, semi-eviscerated weight; SER, semi-eviscerated rate; EW, eviscerated weight; ER, eviscerated rate; BMW, breast muscle weight; BMR, breast muscle rate; AFW, abdomen fat weight; AFR, abdomen fat rate.

**Table 7:** Association of diplotypes of the *PITX2* gene with body size traits in Wuliang Mountain Black-bone chickens (*MEAN ± SE*).

Diplotypes	Body Size Traits							
	BSL (cm)	BW (cm)	BD (cm)	BA (cm)	FBL (cm)	PW (cm)	SL (cm)	SC (cm)
H1H1	22.04 ± 0.66	7.63 ± 0.06	10.98 ± 0.14	55.65 ± 1.12	11.33 ± 0.12	8.48 ± 0.08	10.80 ± 0.12	4.61 ± 0.06
H1H2	21.59 ± 0.15	7.67 ± 0.06	11.26 ± 0.12	54.21 ± 1.08	11.34 ± 0.12	8.42 ± 0.06	10.68 ± 0.10	4.54 ± 0.07
H1H3	21.03 ± 0.32	7.62 ± 0.16	10.87 ± 0.33	60.37 ± 3.14	11.09 ± 0.35	8.71 ± 0.23	10.30 ± 0.27	4.59 ± 0.15
H1H4	21.43 ± 0.16	7.57 ± 0.08	11.11 ± 0.16	56.20 ± 1.47	11.27 ± 0.14	8.56 ± 0.08	10.68 ± 0.27	4.35 ± 0.07^B^
H1H7	22.12 ± 0.20	7.80 ± 0.10	11.29 ± 0.15	56.93 ± 1.46	11.47 ± 0.11	8.59 ± 0.10	10.86 ± 0.11	4.72 ± 0.07^A^
H1H8	21.48 ± 0.48	7.59 ± 0.26	11.70 ± 0.30	55.23 ± 2.32	11.49 ± 0.32	8.35 ± 0.20	10.35 ± 0.24	4.44 ± 0.12
H2H7	21.43 ± 0.28	7.84 ± 0.20	11.22 ± 0.20	54.45 ± 1.82	11.88 ± 0.24	8.42 ± 0.16	10.87 ± 0.14	4.68 ± 0.11
H2H8	21.49 ± 0.46	7.85 ± 0.21	10.86 ± 0.65	50.90 ± 1.83	11.34 ± 0.18	8.37 ± 0.09	10.24 ± 0.47	4.18 ± 0.18

BSL, body slope length; BW, breast width; BD, breast depth; BA, breast angle; FBL, fossil bone length; PW, pelvis width; SL, shank length; SC, shank circumference. Means with different lowercase superscripts differed significantly (*p* < 0.05); means with different capital superscripts differed highly significantly (*p* < 0.01); means with the same letter did not differ significantly (*p* > 0.05).

**Table 8:** Association of diplotypes of the *PITX2* gene with carcass traits in Wuliang Mountain Black-bone chickens (*MEAN ± SE*).

Diplotypes	Carcass Traits										
	LW (g)	CW (g)	SR (%)	SEW (g)	SER (%)	EW (g)	ER (%)	BMW (g)	BMR (%)	AFW (g)	AFR (%)
H1H1	1662.14 ± 29.93	1534.27 ± 28.92	92.21 ± 0.20	1322.23 ± 24.43	79.55 ± 0.38	1080.85 ± 19.15	65.14 ± 0.42	169.70 ± 3.35	15.83 ± 0.25	43.54 ± 3.69	3.61 ± 0.27
H1H2	1665.86 ± 26.81	1528.62 ± 25.25	91.74 ± 0.30	1328.76 ± 22.09	79.76 ± 0.35	1093.57 ± 18.11	65.68 ± 0.37	162.14 ± 4.30	14.84 ± 0.31^B^	38.97 ± 3.23	3.22 ± 0.24
H1H3	1738.53 ± 63.69	1610.16 ± 59.12	92.62 ± 0.49	1386.58 ± 58.00	79.56 ± 0.94	1127.16 ± 46.11	64.73 ± 0.77	166.16 ± 8.45	14.77 ± 0.67	44.18 ± 9.09	3.39 ± 0.62
H1H4	1685.21 ± 35.47	1553.83 ± 35.61	92.05 ± 0.33	1347.94 ± 29.81	79.95 ± 0.38	1115.46 ± 23.89	66.27 ± 0.55	174.84 ± 5.35	15.67 ± 0.34	40.53 ± 5.14	3.23 ± 0.36
H1H7	1766.11 ± 43.94	1611.82 ± 40.67	91.25 ± 0.42	1410.15 ± 34.83	79.94 ± 0.44	1156.16 ± 26.94	65.69 ± 0.44	172.51 ± 4.96	15.20 ± 0.43	46.85 ± 5.99	3.44 ± 0.36
H1H8	1674.20 ± 50.73	1531.80 ± 47.00	91.53± 0.81	1325.80 ± 40.86	79.28 ± 1.04	1094.00 ± 34.22	65.45 ± 1.09	152.57 ± 7.86	13.97 ± 0.67^B^	37.30 ± 7.31	3.10 ± 0.51
H2H7	1715.97 ± 39.59	1566.29 ± 38.20	91.33 ± 0.82	1345.97 ± 33.61	79.08 ± 0.92	1108.17 ± 26.41	65.20 ± 0.87	176.04 ± 6.83	15.91 ± 0.50	38.54 ± 5.65	2.96 ± 0.39
H2H8	1662.80 ± 80.43	1541.70 ± 78.93	92.61 ± 0.53	1376.50 ± 70.44	82.71 ± 0.59	1140.50 ± 61.97	68.46 ± 0.92	201.86 ± 10.57	17.74 ± 0.29^A^	51.19 ± 10.43	4.14 ± 0.76

LW, live weight; CW, carcass weight; SR, slaughter rate; SEW, semi-eviscerated weight; SER, semi-eviscerated rate; EW, eviscerated weight; ER, eviscerated rate; BMW, breast muscle weight; BMR, breast muscle rate; AFW, abdomen fat weight; AFR, abdomen fat rate. Means with different lowercase superscripts differed significantly (*p* < 0.05); means with different capital superscripts differed highly significantly (*p* < 0.01); means with the same letter did not differ significantly (*p* > 0.05).
